# Identifying solutions to meet unmet needs of family caregivers using human-centered design

**DOI:** 10.1186/s12877-022-02790-5

**Published:** 2022-02-02

**Authors:** Vittavat Termglinchan, Samira Daswani, Paricha Duangtaweesub, Taweevat Assavapokee, Arnold Milstein, Kevin Schulman

**Affiliations:** 1 Clinical Excellence Research Center, Department of Medicine, Stanford School of Medicine, Room 326, 366 Galvez St, Stanford, CA 94305 USA; 2 Hasso Plattner Institute of Design (d.school), Stanford, CA USA; 3 Department of Medicine, Faculty of Medicine Ramathibodi Hospital, Mahidol University, Bangkok, Thailand

**Keywords:** Informal caregiving, Family caregiver, Caregiving needs, Culturally sensitive, Independent aging

## Abstract

**Background:**

Given the rapidly aging society, shrinking workforce, and reducing dependency ratio, there is an increasing challenge for family members to provide care for older adults. While a broad understanding of caregiver burden and its consequences have been studied across various contexts, there is a need to better understand this challenge among family caregivers in Asian societies.

**Methods:**

This study is a cross-sectional observational study. A total of 20 dyads of community-based older adults, who required assistance with at least one activities of daily living, and family caregivers in Thailand participated in the study. We used the first three stages out of five stages of human-centered design: empathize, define, and ideate.

**Results:**

On average caregivers were 59.2 years old, with 43% still employed. Of the older adult participants, 10 were interviewed, the others had moderate-to-severe cognitive impairment. Based on the analysis, six caregiver personas (i.e. semi-fictional characters) are identified. Caregiver personas of “The 2-Jober” and “My Life Purpose” has the highest caregiver burden score whereas “The Spouse” has the lowest. Based on the specific needs of the caregiver persona “My Life Purpose”, the team brainstormed more than 80 potential solutions which were classified into three categories of solutions that satisfied the metrics of desirability, feasibility and viability: distributed medical care system, technology-charged care network, and community gathering for rest and recuperation.

**Conclusions:**

These solutions are culturally sensitive given that they are built around established behavioral patterns. This is an illustration of a method of innovation that can be applied to bring a culturally specific understanding, and to develop products and services to enable further independent aging.

**Supplementary Information:**

The online version contains supplementary material available at 10.1186/s12877-022-02790-5.

## Background

Asia is on track to having the oldest population in the world over the next few decades. Japan, South Korea and Thailand lead this demographic shift [1]. As a result, caregiver burden on families will continue to increase, particularly in cultures with societal norms of children and family members caring for their older adults. Economically, these countries will face the joint challenges of a shrinking workforce due to aging as well as the loss in productivity of working caregivers. In just 20 years, other Asian societies will trend towards Japan’s 1:1.5 dependency ratio, where there is only one and a half working individuals to support each older adults [2]. Despite this convergence of demographic and economic factors, our understanding of how to support graceful aging and reduce the burden on the essential informal caregivers remains primitive.

Thailand will be one of the first middle-income countries with universal health coverage to become an “aged” society by 2021 [3], where 20% of the population is over 65 years old. Economically, this will result in a reduction of about 20% of the working population (from 49 million to 40.5 million people) [4]. Thailand represents other Asian markets where a better understanding of the challenges of caregiver burden and independent aging could result in new culturally sensitive products, services and policies could alleviate the impending economic pressures of a rapidly aging society.

### Characteristic of informal caregivers

*Informal* caregivers are unpaid individuals usually within the patient’s immediate community- a spouse, a partner, a family member, a child, friend or neighbor. Almost all Thai older adults live in close proximity to their family- 71% share the same house with their children, 9.4% live adjacent to their children, 7.4% dwell in the same community [5, 6]. As in most Asian countries where the family is responsible for caring for the older adults, 73% of seniors in Thailand receive support for daily activities from their family (spouse, child, sibling, grandchild), and only 5% have paid caregivers [6]. These informal caregivers are the backbone of the aging society.

### Primary caregiver challenges

Caregiver burden is a complex construct with two distinct components: “objective burden” which refers to the tangible impact of caregiving on a caregiver’s life (e.g., financial loss and time lost from daily activities) and “subjective burden” which refers to the caregiver’s perception of burden of care [7, 8].

While a broad understanding of caregiver burden and its consequences have been widely studied across various contexts [9], there is a need to better understand this challenge among informal caregivers within a cultural context, and in light of the locally available resources. Developing culturally astute conceptual frameworks that enable a better understanding of caregiver burden and unmet needs within Asian societies is an understudied area. Hence, this research explores how human-centered design (HCD) can be utilized as a tool to identify solutions to meet the unmet needs of informal caregiver (i.e. family caregivers) while taking into account the user perspectives and local cultural context in Thailand.

## Materials and methods

### Study design

#### Human-centered design

HCD is a methodology for innovation that focuses on identifying the subjects’ needs and prioritizes their pain points while taking into account the local cultural context. HCD is a method that is used to identify and classify solutions for unique segments of the population. It requires an understanding of the problem from the subjects’ perspective, and allows us to identify different perceptions of the same problem across sub-segments of the population [10].

As a methodology, HCD is a collection of techniques that aims to uncover insights about behaviors and motivations, develop solutions based on non-obvious observations, and direct prototyping efforts to address critical assumptions early. HCD has sparked a revolution in product and service development in markets outside of healthcare [10, 11]. The end goal of HCD is to identify the design principles that lead to feasible, desirable, and viable products, services, or policies that best addresses the needs of our study population.

The HCD was chosen for a few reasons. First, HCD is particularly suited for what design theorist Horst Rittel calls wicked problems, “a class of societal problems which are ill-formulated, where the information is confusing, where there are many clients and decision makers with conflicting values, and where the ramifications of the whole system are thoroughly confusing” [12]. The caregiver-older adult relationship plays out in a complex healthcare and social system, representing one such wicked-problem. Second, HCD has a heavy focus on *empathy* that enables developing a deep understanding of the caregiver and older adult dyads leads to a comprehensive yet novel understanding of the problem space. Lastly, it enables the discovery of culturally sensitive and locally relevant needs and insights. The HCD prioritizes in-person, open-ended user interviews to surface discoveries that reflect the user’s life experiences on a given topic in their own environment. In contrast to other methods of primary user research- such as goal-directed design (GDD)- HCD neither limits the line of inquiry to the topic of the research (e.g. older adult caregiving) nor assumes a particular outcome (e.g. asking about resources to give to caregivers to better provide care) [13]. The process instead focuses on identifying the needs or pain points of the user which can then be translated to a point-of-view which reframes the problem for the user that makes sense in their local experience and culture.

The HCD practice has five stages: Empathize, Define, Ideate, Prototype, Test (Fig. S1, Supplementary Material).

1. **Empathize**: The process of understanding the world through the eyes of the users through modes such immersions, interviews and surveys. The goal is to understand a user’s motivations and pain points about a particular product, service, experience, or area of need.
2. **Define**: The synthesis of quantitative and qualitative data discovered during empathy phase into frameworks that capture the user’s pain points. This phase results in an actionable problem statement.
3. **Ideate**: The generation of a large number of ideas that address the problem statement, where previous mental models are disrupted to generate innovative ideas prior to applying any constraints. The goal is to explore the solution space broadly and to generate that extend beyond current constraints.
4. **Prototype**: The translation of an idea into the real world in a low-cost, low-resolution format that the user can interact with. The goal is to translate an abstract concept into a tangible asset.
5. **Test**: This is an iterative step in which users provide their reactions to the prototype. The goal is to iteratively refine the concept capturing the feedback from the users and ensure that their needs are being met.

HCD is an extensive process; therefore, this manuscript will focus on the first three out of five stages of HCD (i.e. empathize, define, and ideate). The empathy stage included data collection, data coding and classifying needs. The define stage included developing caregiver personas, personas attributes and empathy map, and defining problem statements. The ideation stage included brainstorming and identifying solution categories. The two other stages of HCD (i.e. prototype and test) will require additional experiments and are beyond the scope of this study.

### Study population

This study recruited older adults in Thailand who were 65 years of age or older at the time of interview. The older adult participants were required to be dependent on at least one activities of daily living (ADLs) as described in the Barthel index and have family members as their primary caregivers [14].

The number of interviews was based on achieving theoretical saturation, typically between 12 and 20 independent interviews [15]. There were no specific criteria for selecting the caregivers, so the study can explore the unmet needs of family caregivers across various demographics of older adults.

### Data collection

The objective was to observe and understand the behaviors, values and lives of the older adults and family caregivers. Qualitative research helps articulate the complexity of the user experience and embed it within a unique cultural context [9]. Our data collection included three sets of assessments: in-situ structured interview, in-home survey, and 7-day activity diary. Each interaction with each participant is summarized chronologically in (Fig. S2, Supplementary Material).

#### Structured interview

We developed two sets of structured interview guides for the older adults and their caregivers respectively.

The caregiver interview guide had 13 questions, while the older adult interview guide had 11 questions (Appendix 1, Supplementary Material). Both were based around the challenges of performing ADLs and instrumental activities of daily living (iADLs). The semi-structured interviews were conducted in-person in the home with both participants present. One interview question included a “card-sort”, where the researchers individually asked the older adults and the caregivers to rank order ADL or iADL tasks that they need/offer help with. All interviews were conducted in March to June 2019 by native speakers of Thai language, VT and PD.

#### In-home survey

The researchers would request a quick tour of the living space and capture photographs of relevant insights surfaced during the interview e.g. recent modifications to the bathroom and organization of sleeping space.

#### Diary study

This was a task-analysis administering using a structured diary to identify the ADLs where the older adult needed assistance and/or the caregiver supported across each day (morning, afternoon, evening, night) for a period of 1 week. We distributed two sets of 7-day diary study for the older adults and caregivers to complete (Appendix 2, Supplementary Material). Each day, we had the older adults and caregivers assess their individual mood on a 5-point scale. The survey enabled users to record problems as they arise and it’s fresh in their memory [16], while the anonymity and recording personal activities such as toileting, in the absence of the researchers, increases comfort. The key drawback of this approach is the reliance on the user’s ability to read and write.

At the completion of the study, the interviews and diary studies conducted in Thai were professionally transcribed verbatim and translated into English by a third-party team. All analyses reported in this paper were conducted in English.

#### Caregiver burden assessment

Given that there is no validated caregiver burden tool in the Thai language, the 22 item Zarit Burden Interview was professionally translated into Thai for use in this study [17, 18]. It was administered immediately after the caregiver interview.

### Data analysis

#### Data coding

The interviews were coded using the software ATLAS.ti (ATLAS.ti Scientific Software Development GmbH, Berlin, Germany). The grounded theory approach was used to develop codes based on concepts including text words, lines or paragraphs.

#### Classifying needs

The codes were further categorized into themes that were used to identify needs. Two researchers identified themes independently using grounded theory approach through inductive reasoning, and then final themes were defined collaboratively.

There were two broad types of needs that were identified: revealed needs and latent needs. Revealed needs are explicitly observed, often articulated as functional tasks, and common to the caregiving experiences across many participants. Latent needs are more implicitly defined by the participants and may have an underlying emotional aspect. These were typically specific to each persona.

#### Developing personas

A persona is a semi-fictional character based on the individuals interviewed [19, 20]. The persona reflects who the users are, how they behave, what they need, and what might be preventing them from meeting their own needs. Personas are created from a combination of demographic and psychographic data collected in the interviews, surveys and diary studies. Latent needs were utilized to develop personas.

#### Persona attributes

We selected three attributes that best describe caregiving in the context of Thailand: use of technology, level of spirituality, and decision-making role.

##### Use of technology

Use of technology describes a persona’s attitude towards using technologies to assist them in their day-to-day duties as caregivers. In this case, technology broadly refers to computers, online education resources, communication devices, and mobility-related assistance.

On the *technophile* end, we can expect that a persona is fluent in and comfortable using up-to-date technology, such as CCTV for remote monitoring, mobile messenger application to communicate with doctors in real-time, or custom-made walk-assist contraptions. On the *tech averse* end, we can expect that a persona might still contact doctors via phone calls, learn caregiving tips from friends or during doctors’ appointments, or have hardly any caregiving-specific technologies at home.

A neutral ranking on this attribute suggests that the caregiver neither relies on or shy away from new technology. The underlying principle here is that technology is a reflection of a caregiver’s willingness to rely on an external object to ease their burden. This axis indicates to us how reliant we can be on using new modes of technology in delivering care.

##### Level of spirituality

Level of spirituality describes a persona’s attitude towards religion or mindfulness as a lens of framing their caregiving experiences.

On the *spiritual* end, we can expect that caregivers (and some older adult participants) offering food to the monks and visiting temples, and offering prayers at a certain time of day as a routine in the family. They would discuss karma as well reciprocity (i.e. giving back care to family members) in the context of Buddhism. Personally, these caregivers would also practice meditation or breathing techniques as a way to destress. On the pragmatic end, caregivers would think of their care in terms of ADL and iADL needs and would rely on exercise, entertainment, or respite care to destress.

This attribute indicates how we should frame potential solutions, as a convenience or as a ritual approach that helps them feel closer to their older adults.

##### Decision-making role

Decision-making role describes a persona’s role in the household when decisions about caregiving- medical or otherwise- comes up. This attribute indicates to us who we need to engage beyond the caregiver themselves when discussing new prototypes or solutions.

On the *decision-maker* end, we can expect caregivers to take full control of the decision by conversing with doctors, consulting with friends, or through own research. The caregiver can create their own action plans for what to do next. On the *decision-supporter* end, we can expect caregivers to have similar conversations; however, in a role of coordinating information between medical staff and family members, or of consulting with doctors or family in the next best action items for caregiving home.

As with previous works, these attributes are empirically derived from the interviews and can be used to inform downstream processes such as the development of prototypes to satisfy revealed and latent needs. These attributes are qualified on a relative scale, comparative amongst the personas [21, 22].

#### Empathy map

An empathy map is a framework that categorizes what is observed about a user or user persona (what they say and what they do about a subject) and what a research team infers about their motivations (what they think and feel about a subject) into four quadrants. Qualitative data such as direct quotes and activities observed by the research team (“say” and “do”, on the left side of the empathy map) are used to infer something about the users’ implied thoughts and feelings (“think” and “feel”, on the right side of the empathy map). The empathy map is a way to plot out qualitative data points so that we can identify any inconsistencies in their behaviors and motivations (e.g. does what the user say reflect what they do? does what they do oppose what they think they should do?) (Fig. S3, Supplementary Material).

#### Defining problem statements

The problem statement is framed in the form of a “How Might We” statement. This structure, typical of the HCD method, invites the research team to anchor the problem statements in the caregiver’s latent needs. Based on the insights generated from the user personas, attributes and empathy maps, problem statements pertaining to each user persona were developed.

#### Brainstorming solutions

Two multi-hour brainstorming sessions were held to identify solutions for the unmet needs of the caregivers and the older adults. HCD mindset governed the sessions, namely, generating a diverse set of ideas, observing a “yes-and” mindset, visualizing ideas, and encouraging wild, seemingly impossible solutions. The prompts used to generate the brainstorm stemmed from the problem statements defined.

#### Identifying culturally specific solutions

Ideas and solutions were down-selected using the framework of desirability, feasibility and viability. Desirability represented the end user’s unmet needs, feasibility represented the availability to build the solution, and viability represented the ability to build a sustainable business or market for the solution. Ideas and solutions that sat at the intersection of that Venn diagram were prioritized to be candidates for later research.

### Statistical analysis

Baseline characteristics of the two groups were described using proportions for categorical variables and means with standard error of the mean (SEM) for continuous variables. Analyses were performed using SPSS Statistics Subscription (IBM, NY, USA).

## Results

### HCD empathy stage

A total of 20 caregiver-older adult dyads were interviewed. All caregivers completed the interview and the burden survey, while 15 (75%) of them completed the 7-day diary study. On average caregivers were 59.2 years old, with 45% still employed. Of the older adults in the study, 10 were interviewed, the others had moderate-to-severe cognitive impairment (Table 1).

**Table 1:** Caregiver and older adult demographics

Characteristics	Result
Caregiver characteristics	
Total caregivers^a^, n	20
Burden survey completed, n (%)	20 (100)
Diary study completed^b^, n (%)	17 (85)
Sex	
Male, n (%)	3 (15)
Female, n (%)	17 (85)
Age, mean (SD), y	59.2 (9.2)
Employment status	
Employed, n (%)	9 (45)
Retired, n (%)	11 (55)
Relationship to older adults	
Spouse, n (%)	3 (15)
Sibling, n (%)	1 (5)
Children, n (%)	16 (80)
Average number of ADLs assistance (out of 5), n (range)	2.3 (1–5)
Average number of iADL assistance (out of 9), n (range)	4.3 (1–9)
Zarit Burden Interview, mean (SD)	21.6 (11.3)
Older adult characteristics	
Total older adults, n	20
Total older adults interviewed, n (%)	10 (50)
Sex	
Male, n (%)	6 (30)
Female, n (%)	14 (70)
Age^c^	
Age, mean (SD), y	86.2 (7.2)
Family structure	
Single, n (%)	1 (5)
Divorced with children, n (%)	2 (10)
Married with children, n (%)	12 (60)
Widowed with children, n (%)	5 (25)

^a^Some families have multiple caregivers, including family and paid caregivers who support in secondary roles, and primary caregivers who split responsibility equally. Secondary and paid caregivers are not included in this table

^b^Only one set of diary studies are assigned per family, regardless of how many primary and secondary caregivers are present

^c^Two participants did not disclose their age

A master set of codes, associated with phrases that represent qualitative data or insight into possible needs from the interviews, were created. In total, 158 codes were identified, highlighting specific quotes from each participant. Table S1 in Supplementary material demonstrates the top five most frequently identified codes from the interviews.

The codes were further categorized into themes that were used to identify needs: revealed needs and latent needs. Revealed needs, easier to identify, are associated directly with an interview question. For instance, the theme of “methods of de-stressing”, is in direct response to the question: “what do you do when you feel low?”. The code set is therefore grouped around stress, relief, support from family and friends (Table S2, Supplementary Material).

We found four basic categories of revealed needs: de-stressing habits, respite care, education, and access to mental health care (Table S3, Supplementary Material). Many of participants found a workaround to address some of these needs. For example, 15 out of 20 people are able to relieve stress through small habits such as calling up their relatives to talk when they have problems at home. Though each participant can share the same needs, each might manifest different solutions to address them.

Latent needs rely on the researcher’s assessment of the user’s attitudes that became explicit during the interview. A caregiver who took an older adult back home from the nursing home is an example of a duty-based commitment to the role of caregiving. As such, latent needs associated with each user persona are less directly derived from the broad themes of the codes; instead they are based on what the caregiver said, did, thought of, and felt and inferred their latent needs (Table S4, Supplementary Material).

### HCD define stage

Table 2 demonstrates six broad categories of latent needs for caregivers each indicating a unique user persona. While each persona demonstrates similar revealed needs with each other, latent needs remain largely unmet and are the key emotional pain points of caregiving. Caregiver personas of “The 2-Jober” and “My Life Purpose” have the highest caregiver burden score whereas “The Spouse” has the lowest score. User personas were developed only for the caregivers, as many of the older adult subjects we interviewed had severe cognitive impairment and were not able to communicate during the interviews. In addition, these personas were compared and ranked across the three attributes: use of technology, level of spirituality, and decision-making role (Fig. S4, Supplementary Material).

**Table 2:** Latent needs observed across six user personas of caregivers, in descending order of burden score

Persona	Latent Need	Description	Total Participants	Average Burden Score
The 2-Jober	Needs to maintain constant communication with the older adults to feel less guilty while at work	This persona walks a fine balance between job and caregiving as two full-time jobs	1	44.0
My Life Purpose	Needs to less guilty for taking respite care	This persona takes pride in taking care full-time, as the sole coordinator and caregiver	7	27.7
The Entrepreneur	Needs convenience when obtaining and using solutions for care	This persona is self-employed and thus flexible in their time. They appreciate getting things done, fast.	2	20.0
The Informal Nurse	Needs to access formalized medical training to feel at ease about role	This persona is the default choice in the family to take care of the older adults, though relegates medical decisions to someone else	5	17.4
The Improviser	Needs support across all aspects of care; medical, physical, and mental	This persona finds themselves doing everything in caregiving regardless of expertise	3	14.3
The Spouse	Needs to feel supported by their family members in caregiving	This persona is the spouse who also has their own needs similar to those of the older adults they’re taking care of.	2	11.5

Next, two criteria, high burden score and certainty of need, were used to down-select personas These personas would constitute people who we can be most sure have the most burdensome need**.** “My Life Purpose” persona, whose need is “to feel less guilty for taking respite care”, has a high occurrence with a high caregiver burden score (Fig. S5, Supplementary Material). Therefore, it will be used to demonstrate the use of in-depth design tools to come up with novel and human-centric ideas to prototype.

**Fig. 1:**
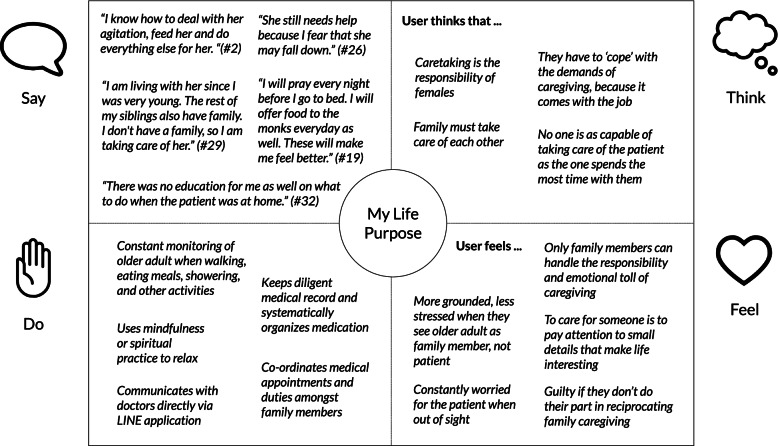
An empathy map framework describing the user persona “My Life Purpose”. The left quadrants (“say” and “do”) aggregates the observed communications and behaviors of the participants who belongs to the “My Life Purpose” persona, while the right quadrants (“think” and “feel”) are implied thoughts and feelings about their caregiving experience. The empathy map organizes qualitative raw data to show the characteristics of single user persona (a group of users who share the same latent need)

Figure 1 shows the empathy map of “My Life Purpose” persona. Specifically, this persona devotes a lot of time to coordinating care and monitoring the older adults as well as practices spiritual or mindfulness rituals to relax. In addition, shared beliefs and emotions such as gender norms that females are more suited to caregiving, and feelings of guilt as a mode of motivation to care for their family (indicated by the “think” and “feel” quadrants) are exhibited.

### HCD ideate phase

**Fig. 2:**
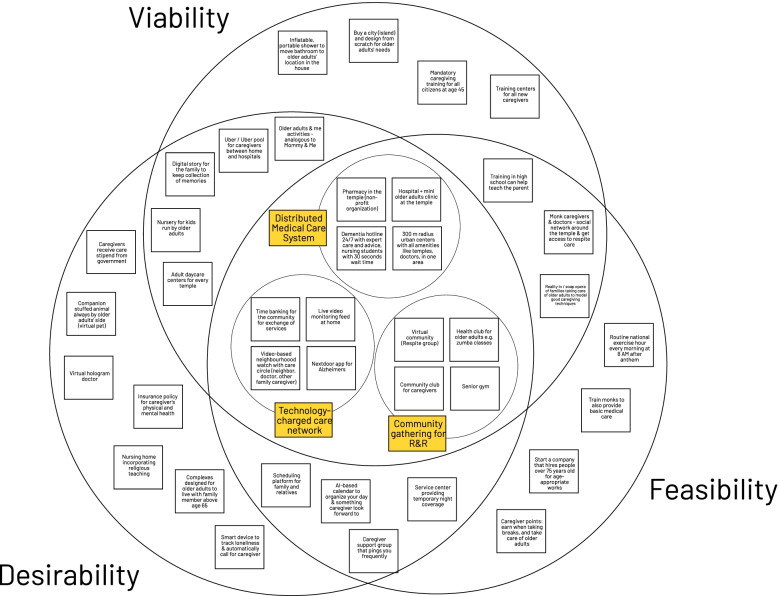
Desirability, viability, and feasibility model is a subjective framework for down-selecting ideas. These ideas are subjective and based on the experience and knowledge of the research team; hence, the importance of an interdisciplinary team for HCD process

Subsequently, we defined problem statements for “My Life Purpose”, such as “How might we help the “My Life Purpose” feel less guilty about taking time for themselves?”. Based on the problem statement, the research brainstormed solutions to address the unmet needs of “My Life Purpose” persona. A total of 82 ideas were generated from this process. These ideas were clustered into different themes (e.g. “community-based initiatives”, “leveraging religious network”, and re-design infrastructure”) (Fig. S6, Supplementary Material), and then were down-selected based on the based on the criteria of desirability, viability, and feasibility into three potential solutions: a distributed medical care system, technology-charged care network, and community gathering for rest and recuperation (R&R) (Fig. 2).

## Discussion

Given the rapidly aging society, shrinking workforce, and reducing dependency ratio in Asian societies, there is an increasing need for family members to provide care for older adults. Yet, there hasn’t been much study to better understand the unmet needs of family caregivers in most Asian countries, including Thailand.

In previous studies on older adult care in Thailand, there have been minimal attempt to directly link the insights from the descriptive findings to potential interventions in a single connected process [23] Here, we have shown that HCD can be utilized as a comprehensive framework to identify caregiver’s unmet needs, define problem statements and identify culturally sensitive solutions. Previous studies using HCD in healthcare are not focused on the unmet emotional needs of family caregivers, and tends to focus on the user’s revealed needs related to functional tasks or products, e.g. motivation to use smart home technology [24], relationship to consumer health technology [22], amount of time spending with older adults [21], digital health advisor [25], or frequency and type of symptoms of patients in an emergency department [26]. In our study, we have anchored our personas specifically to the family caregivers, and have focused on the latent needs; thus, making the insights cultural-sensitive and product-agnostic.

Our research demonstrates the role of the family and community in caring for the older adults in an aging Asian society. We found that the typical primary family caregivers in Thailand are supported by a number of secondary family caregivers and the community itself (i.e. friends, neighborhood and broader societal community). These findings add nuance to what has been previously known about the multi-generational household structure of caregiving in Asian societies [5, 6, 27].

**Fig. 3:**
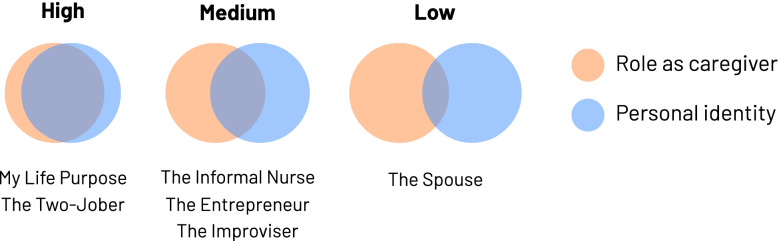
Venn-like diagrams with the overlapping circles of “personal identity” and “role of caregiver”. This diagram indicates the extent to which the caregiver identifies as a caregiver, rather than having a familial role (i.e. child, sibling, or spouse)

There are four common areas of unmet revealed needs that were identified across the family caregivers. These include the need for de-stressing habits, respite care, caregiver education, and fall prevention. However, it is important to note that there are unique needs, motivations and characteristics of caregiving based on the personas of the caregivers. We developed six caregiver personas. Each persona, based on demographic, psychographic and behavioral markers, showcases distinct objective and subjective degrees of burden and burnout. Caregiver personas with high burden scores are “The 2-Jober” and “My Life Purpose” whereas “The Spouse” has the lowest burden score. This finding may be explained by the caregiver identity theory which suggests several factors related to the development of family caregiver identity including loss of shared identity (i.e. being perceived as a caregiver rather than a familial member) [28] (Fig. 3).

The HCD analysis, including empathy mapping and personas attributes, of each persona demonstrates unique characteristics which support why specific solutions may be more effective for certain families. For example, some caregiver personas are more tech averse, pragmatic, and decision-making than the others. Our research found that older adults in Thailand significantly rely on assistive devices and technologies to support their daily life; however, their exhibited behaviors vary. Some families will purchase available assistive devices, while some will create their own tools at home. Therefore, solutions will have to be personalized to each user persona in order to be truly effective. Leveraging already existing behavioral attributes would enable rapid adoption of interventions.

Based on the latent need of the “My Life Purpose” persona, the team brainstormed more than 80 solutions. Three groups of ideas were identified that satisfied the metrics of desirability, feasibility and viability: distributed medical care system, technology-charged care network, and community gathering for R&R. As an illustration of why HCD-based solutions are culturally sensitive, we can see these solutions involve local community centers such as the temples, which is an essential part of Thai people’s lives [29]. Temples have a close relationship with all classes of Thai people, in terms of religious, social and education issues. Currently there are about 40,000 temples in Thailand, roughly about the same number of schools in the whole country.

The limitation of this research largely lies in its ability to be generalized across all countries as this study was conducted in Thailand. However, the HCD can be adopted in other health system research. In addition, our research was focused mainly on identifying the unmet needs of family caregivers in an aging society; therefore, the results may only be applicable in similar cultural and societal systems. Lastly, since there was no validated version of Zarit burden interview in Thai language, the results may only be used within this study, but not to be compared with previous studies.

## Conclusions

There are latent needs of older adult and caregiver pairs in the aging society and utilizing HCD approach enhanced with quantitative tools of ethnographic coding, surveys and diary studies in health system research is an innovative framework to better understand the problems and identify solutions that are specific, effective, and culturally-sensitive to sub-groups of the population.

## Supplementary Information

**Additional file 1.**

## Data Availability

All data generated or analysed during this study are included in this published article and its supplementary information files.

## Abbreviations

HCD: Human-centered design
ADL: Activities of daily living
iADL: Instrumental activities of daily living
GDD: Goal-directed design
R&R: Rest and recuperation

## References

[1] Ann. How Asia’s population is aging, 2015–2030 scenario (2018). The Jakarta Post.

[2] The World Bank. Age dependency ratio (% of working-age population). Age dependency ratio (% of working-age population). Available from: https://data.worldbank.org/indicator/SP.POP.DPND?end=2018&start=2018&view=map

[3] Sarmiento P. Thailand set to face growing demands of elderly transition. Hong Kong: China Daily; 2019. Available from: www.chinadaily.com.cn/a/201903/21/WS5c934b18a3104842260b1d7a.html

[4] Kongrukgreatiyos K. Thailand’s Economy Expected to Grow 2.5 Percent in 2016. Bangkok; 2016. Available from: https://www.worldbank.org/en/news/press-release/2016/06/28/thailand-economic-monitor-june-2016

[5] Knodel J, Chayovan N (1997). Family support and living arrangements of Thai elderly. Asia Pac Popul J..

[6] The Changing Well-being of Thai Elderly. 2011. Available from: https://www.helpage.org/silo/files/the-changing-wellbeing-of-thai-elderly-an-update-from-the-2011-survey-of-older-persons-in-thailand.pdf

[7] Gater A, Rofail D, Tolley C, Marshall C, Abetz-Webb L, Zarit SH (2014). “Sometimes It’s difficult to have a normal life”: results from a qualitative study exploring caregiver burden in schizophrenia. Schizophr Res Treatment.

[8] Knight BG, Robinson GS, Flynn Longmire CV, Chun M, Nakao K, Kim JH (2002). Cross cultural issues in caregiving for persons with dementia: do familism values reduce burden and distress?. Ageing Int.

[9] Gray RS, Hahn L, Thapsuwan S, Thongcharoenchupong N (2016). Strength and stress: positive and negative impacts on caregivers for older adults in Thailand. Australas J Ageing.

[10] Lewrick M, Link P, Leifer L. The design thinking playbook: mindful digital transformation of teams, products, services, businesses and ecosystems. USA: Wiley; 2018. Available from: https://books.google.com/books?id=jMlZDwAAQBAJ

[11] Brown T (2008). Design thinking.

[12] Rittel HWJ, Webber MM (1973). Dilemmas in a general theory of planning. Policy Sci.

[13] Fore D, Goldenhar LM, Margolis PA, Seid M (2013). Using goal-directed design to create a novel system for improving chronic illness care. JMIR Res Protoc.

[14] Mahoney FI, Barthel DW (1965). Functional evaluation: the BARTHEL index. Md State Med J.

[15] Saunders B, Sim J, Kingstone T, Baker S, Waterfield J, Bartlam B (2018). Saturation in qualitative research: exploring its conceptualization and operationalization. Qual Quant.

[16] Israsena Na Ayudhya P, Piromya P, Boonvong N (2007). CAREGIVING FOR THE ELDERLY IN THAILAND.

[17] Seng BK, Luo N, Ng WY, Lim J, Chionh HL, Goh J (2010). Validity and reliability of the Zarit burden interview in assessing caregiving burden. Ann Acad Med Singap.

[18] Zarit S, Orr NK, Zarit JM. The hidden victims of Alzheimer’s disease: families under stress. USA: NYU Press; 1985. Available from: https://books.google.bj/books?id=RtoVCgAAQBAJ

[19] Perfetti C (2007). Goal-directed design: an interview with Kim Goodwin.

[20] Chapman C, Milham R (2014). The personas’ new clothes: methodological and practical arguments against a popular method.

[21] Al Awar Z, Kuziemsky C (2017). Persona development and educational needs to support informal caregivers. Stud Health Technol Inform.

[22] LeRouge C, Ma J, Sneha S, Tolle K (2013). User profiles and personas in the design and development of consumer health technologies. Int J Med Inform.

[23] Caffrey RA (1992). Caregiving to the elderly in Northeast Thailand. J Cross Cult Gerontol.

[24] Burrows A, Gooberman-Hill R, Coyle D (2015). Empirically derived user attributes for the design of home healthcare technologies. Pers Ubiquit Comput.

[25] Bhattacharyya O, Mossman K, Gustafsson L, Schneider EC (2019). Using human-centered design to build a digital health advisor for patients with complex needs: persona and prototype development. J Med Internet Res.

[26] Jacob R, Wong ML, Hayhurst C, Watson P, Morrison C (2016). Designing services for frequent attenders to the emergency department: a characterisation of this population to inform service design. Clin Med (Lond).

[27] Subgranon R, Lund DA (2000). Maintaining caregiving at home: a culturally sensitive grounded theory of providing care in Thailand. J Transcult Nurs.

[28] Eifert E, Adams R, Dudley W, Perko M (2015). Family caregiver identity: a literature review. Am J Health Educ.

[29] Sethabouppha H, Kane C (2005). Caring for the seriously mentally ill in Thailand: Buddhist family caregiving. Arch Psychiatr Nurs.

