# Supplementary material for: Identifying solutions to meet unmet needs of family caregivers using human-centered design
Source: BMC Geriatr. 2022 Feb 2;22:94. doi: 10.1186/s12877-022-02790-5 (PMC8812059; doi:10.1186/s12877-022-02790-5)
Supplement: Supplementary file 1 — Additional file 1. [file 12877_2022_2790_MOESM1_ESM.docx]

**Identifying solutions to meet unmet needs of family caregivers using human-centered design**

**SUPPLEMENTARY MATERIAL Page**

**Supplementary Figures**

**Figure S1 2**

**Figure S2 3**

**Figure S3 4**

**Figure S4 5**

**Figure S5 6**

**Figure S6 7**

**Supplementary Tables**

**Table S1 8**

**Table S2 9-10**

**Table S3 11**

**Table S4 12-15**

**Appendix 1: Interview guides for older adults and caregivers 16-19**

**Appendix 2: Diary study for older adults and caregivers 20-22**

**Appendix 3: Master code list 23-28**

**References 29**

**SUPPLEMENTARY FIGURES**

**Figure S1:** 5-stage process of human-centered design (HCD) was developed by the Hasso-Plattner Institute of Design at Stanford University (d.school). The d.school is considered the global leader in both teaching and refining the steps of design thinking.

**Figure S2:** Data collection process.

**Figure S3:** Empathy map framework that categorizes what is observed about a user or user persona into four quadrants (what they say and what they do about a subject- left quadrants) and what a research team infers about their motivations (what they think and feel about a subject- right quadrants).

**Figure S4:** User personas attributes show the characteristics of each user persona on a relative scale, defined by use of technology (technophile/tech-averse), level of spirituality (spiritual/pragmatic), and decision-making role in the household (supporter/maker). These characteristics can guide possible ideas for intervention and help us and differentiate the type of interventions that are most likely to work with each persona. For example, we can suggest possible interventions at a place of workshop (e.g. temple) for the highly spiritual “My Life Purpose”, or technology-light interventions for communicating with physicians for “The Spouse” or “The 2-Jober”.

**Figure S5:** Plot showing the average caregiver burden score of each user persona as determined by translated Zarit burden survey (score ranges from 0 to 88) and the number caregivers (n) interviewed in the study that can be categorized under the same user persona (i.e. sharing the same latent need).

**Figure S6:** The results of open-ended ideation based on a How Might We statement generated from latent need of My Life Purpose persona, clustered into similar themes for further analysis using desirability/feasibility/viability model.

**SUPPLEMENTARY TABLES**

**Table S1:** The top five most frequently identified codes and their examples

| Code | Example Quote | Frequency |
| --- | --- | --- |
| Living Situation | *“3 people living in this house: my 2 children and me. The house close by is my sister's (the patient). And the farthest house is my oldest son’s”* | 83 |
| Medication Management | *“I was giving the patient one medication and she didn't want to swallow; I asked the doctor what to do. He told me that it could be crushed, so now that patient is able to take it.”* | 76 |
| Older adult - Medical Care | *“Six months passed, she was very happy at the nursing home. However, every time that I visited her, she always cried and would like to come back with me. That’s when I brought her back to my house again.”* | 71 |
| Dementia/ Memory | *“When she was transferred to work in the office instead of teaching, she was unable to calculate money.”* | 69 |
| Mobility | *“When the patient walks, she will feel tired, and she doesn't know how to stop and take a break.”* | 67 |

**Table S2:** An example codes grouped into themes to identify revealed needs

| **Theme** | **Codes Set** | **Example of Quote** |
| --- | --- | --- |
| Having established methods of de-stressing helps sustain the caregiver | Caregiver - health  Caregiver friends  Community centered  Reducing burden  Caregiver - technology  Temporary relievement  Caregiver affect - stress  Caregiver affect - relief  Emotional exhaustion  Meditation  Cultural norms - religion  Entertainment | "I just stop, have some food, and lay down to watch TV." (#14)  "I will talk with my friends. Even I am retired from work, I still keep in touch with my friends. There are several groups of friends: friends from school and university." (#26)  "I will do gardening and feed the fish … I will pray every night before I go to bed. I will offer food to the monks everyday as well. These will make me feel better." (#19)  "I will run and read books. " (#29) |
| Need for educational support on how to care is essential. Currently caregivers rely on informal channels through online videos & tutorials | Caregiver - medical learning  Caregiver affect - helpless  Caregiver - education  Older adult - medical care  Home care  Resources - education  Paid caregivers  Caregiver affect - worry  Caregiver affect - responsible | "I received training from [the hospital]. It was a 2-day training for taking care of older adults and patients with dementia. I forgot most of the teaching now." (#26)   "My father was sick for a while, so I watched how the paid caregiver took care of him." (#15)  "I used to be a nurse, so I didn't receive any specific training. I learn new things from doing.” |
| Respite care typically provided from an additional family caregiver or a paid caregiver | Respite care  Caregiver - family support  Temporary relievement  Caregiver responsibility  Caregiver family  Caregiver affect - stress  Paid caregiver  Caregiver - sleep  Care coordination  Medical management  Caregiver affect - worry  Housekeeping | "Sometimes when my sisters knew that I was tired, we would go to a café for about 2-3 hours. This helps relieve my stress." (#10)   "I can't stay with my mother all the time. I also have my own family, and I need someone who can stay with my mother 24/7. " (#16)  "Only when my older sister is home, I will go back to my home. I will be here most of the weekdays. My sister usually returns home on weekend." (#15) |
| Fear of falls is a common worry for caregivers | Caregiver affect - fear  Caregiver affect - worry  Bathing  Showering  Toileting  Falls  Caregiver burden  Caregiver friends  Network  Pets  Paid caregivers  Dressing  ADL | "I am afraid that she may slip and fall in the restroom. I have to watch her during bathing and toileting" (#5)   "I have to help her and stay with her. She can walk but I am worried that she may fall. I have to observe her all the time." (#14)   "There is one dog which likes to jump at the patient. I am afraid that he will fall." (#35) |

**Table S3:** Revealed needs commonly observed across caregivers

| Revealed Need | Description | Occurrence (out of 20) |
| --- | --- | --- |
| De-stressing habits | Small habits practiced regularly help the caregivers relieve stress and continue to work in their role  *For example: sharing their feelings with relatives over the phone* | 14 |
| Caregiver education | For caregivers with non-medical backgrounds, learning about older adult care-specific practices help the caregivers adjust to their new responsibilities  *For example: learning about mental disease help caregiver better communicate with her mother* | 12 |
| Respite care | A dedicated time-off from their duties help caregivers to recharge, away from their family members  *For example: two siblings alternating holidays* | 11 |
| Fall prevention | The biggest source of concern is potential injury from falling, while playing with pets, toileting, showering, between floors, while getting dress, or moving around the house.  *For example: feeling afraid to leave patients during showers or toileting* | 8 |

**Table S4:** An example codes grouped into themes to identify latent needs, which are inferred from the participant’s words, actions, feeling, thoughts

| **Theme** | **Latent Need** | **Codes Set** | **Example of Quote** |
| --- | --- | --- | --- |
| Feelings of guilt underlying caregiving | Needs to less guilty for taking respite care  [My Life Purpose] | Nursing home  Quality of care  Caregiver affect - worry  Dementia/Memory  Showering  Caregiver responsibility  Caregiver affect - “Not enough”  Caregiver affect - responsible  Caregiver affect - validation | “I randomly went [to the nursery] once without telling the staff to check on the patient. They took care of her very well.” (#2)  “I [have been] living with her since I was very young. The rest of my siblings also have family. I don't have a family, so I am taking care of her.” (#29)   “I used to have an aunt who took care of me since I was young. She passed away from cancer when I was working. I felt so bad that I didn't have a chance to take care of her prior to her death.” (#15) |
| Juggling responsibilities between caregiving, work, and personal time | Needs to maintain constant communication with the older adults while having clear demarcation of duties and time  [The 2-Jober] | Care coordination  Caregiver - medical learning  Caregiver family | "Each month there will be appointments with cardiologist, neurologist, and other doctors. My brother and I will rotate to take my father to the appointments. … After I have taken him to several appointments, I learn more and more on how to take care of the patient. But sometimes I can't take care of him because I also have my own work to do." (#24)   "I am married, but I don't have children. Like I told you, my father doesn't want me to go anywhere. If he is not around, I may already have a child. I have to manage my time to take care of my father and spend time with my husband. I am married for 20 years and work at the same place with my husband." (#24) |
| In position of making care decisions, but feels out of their depths | Needs support across all aspects of care; medical, physical, and mental  [The Improvisor] | Dementia / Memory  Caregiver - education  Caregiver affect - frustration  Caregiver affect - stress  Caregiver - medical learning  Caregiver - health Medical care  Caregiver burden  Caregiver affect - acceptance  Preparing meals  Caregiver friends  Network  Caregiver - family support  Caregiver - sleep  Older adult - medical care  Emotional care | "My friend's sister advised me. His/her mother also has dementia as well." (#25) - on seeking care advice  "I used to be mad that I have to wake up early to take her to see the doctors. It really depends on your perspective… I don't really care how much I can sleep every night now. Sometimes she will call me almost every hour at night because she wants to use the restroom, but she doesn't know where it is." (#9)  "Even the doctor… told me that I don't really understand the patient. He mentioned that … I have to realize that she is older and also has Alzheimer's. Therefore, she may not do things that I tell her to do. When I mentioned to her not to do things, she would still do them over and over again. " (#10) |
| Inhabit whatever role is needed of them, based on own knowledge or informal learning | Needs to access formalized medical training to feel at ease about role  [The Informal Nurse] | Older adult - medical care  Caregiver employer status  Caregiver responsibility  Decision making  Emergency  Caregiver - education  Caregiver - family support  Care coordination  Resources - education  Care coordination  Feeding  Medication Management  Older adult - agitation, panic  Older adult - combative  Caregiver affect - responsible | “They used to call me when I was working. I also have to switch my shifts to take my parents to see the doctors. Some colleagues didn't understand this, so I have to retire from work. I am the main person to take them to the appointments.” (#30)   "Most of the time, I will be the one who makes this decision. But I will also ask other family members as well. We will try to discuss and choose the one that everyone agrees.” - CAREGIVER #2 (Caregiver #1 is the main primary one who stays with patient) (#20)  “The patient has 9 children. I am the 5th, and I am a nurse. I have 5 siblings who are doctors. My 2nd sister is an internal medicine doctor so she will make decisions on his care.” (#22)   “We will read about behaviors that are associated with older patients. We didn't read much on how to take care of them." (#34)   "When mom returned home, I was able to help suction her sputum for a week, then I had to stop because she strongly refused and also bit my fingers." (#7) |
| Approaches caregiving with practicality | Needs convenience when obtaining and using solutions for care  [The Entrepreneur] | Older adult independence  Older adult isolation  Grooming  Resources - education  Caregiver affect - helpless  Care coordination  Respite Care  Caregiver isolation  Caregiver family  Caregiver responsibility  Mobility  Falls  Caregiver - family support  Caregiver affect - guilt  Paid caregivers  Home modifications  Older adult independence  Caregiver affect - sad  Caregiver affect - patience  Quality of care  Remote care  Caregiver affect - care Caregiver Demographics  Older adult - finances  iADL - finances  Older adult - dignity  Emotional exhaustion  Preparing meals  Caregiver affect - calm  Caregiver affect - responsible  Caregiver affect - relief | "She doesn't comb her hair anymore. Before we didn't have her hair cut short, but because she couldn't care for this herself, so I had it cut. It is easier to be cleaned now." (#14)  "I am her son, so I don't feel disgust with her urination, defecation or vomitus. When I show my family that I am comfortable to clean her, everyone else will feel comfortable to take care of her as well." (#16)   "I will provide her a wallet when she is going outside such as going to the church. She can manage her own spending there. However, when she returns home, I will take her wallet and store it for her. She used to get worried about where her wallet was, and tried to look for it all day" (#33)  "I just talked with the caregiver and she will ask my mom to help prepare the ingredients such as preparing vegetables. ... I don't want her to stay still. We want to find activities for her." (#33) |
| Relying on family members to help out with everyday activities, resources | Needs to feel supported by their family members in caregiving  [The Spouse] | Pets  Living situation  Proximity to family  Transportation  Caregiver - family support  Caregiver responsibility  Caregiver - health  Peace of mind  Co-dependency  Care coordination  Care financing  Older adult - happiness | “Yes, his daughter invited us to live here. She wants us to help take care of the house. She doesn't trust other people.” (#35) - on their extra responsibility to take care of the current house  “I will also tell my children and grandchildren that we will go to the hospital. They can communicate better with the staff there. I think I am from the older generation so my communication may not be understandable with the hospital staff.” (#5) |

**APPENDIX 1: Interview guides for older adults and caregivers**

**Interview Guide for** **caregivers**

What is your relationship with the older adult/family?

3-PART INTERVIEW

● Part 1: Act of Caregiving

● Part 2: Life as Caregiver

● Part 3: Context of Care

**Part 1: Act of Caregiving**

1.  How many hours per day are you caring for this person?

2.  What kind of activities do you help the older adult with?

3.  Of these cards, which ones are you helping with? Of the ones you are helping with,

name the top 3 that are challenging, and the 3 easiest. Explain why. (For ease of

asking the question, we shall show them a cards with each of the words).

a) ADLs

i) Bathing

ii) Eating

iii) Getting dressed

iv) Toileting

v) Transferring

vi) Continence

b) iADLs

i) Medication management

ii) Pet care

iii) Managing finances

iv) Preparing meals

v) Shopping

vi) Driving or managing other forms of transportation

vii) Home maintenance

viii) Cleaning and laundry

ix) Using communication devices such as a phone

4.  How did you learn how to help with these activities?

5.  What aspects of their care worries you the most?

6.  In case of emergencies, what do you do?

7.  Please describe the most recent encounter with a medical professional. Why? What

happened? Any recent hospitalizations?

**Part 2: Life as Caregiver**

8.  What resources have you used in caring for them that have been the most helpful?

(financial, equipment, time, paid help, etc.)

9.  Who supports for you?

10.  (If they are employed) - When was the last time you had to take time off from work to

care? How often does that happen?

11.  What gives you energy when you are feeling low?

**Part 3: Context of Care**

12.  When was the last time you moved homes? If yes, then why? If applicable, how long

does it take for you to travel here?

13.  Can you tell us about the space? How did you organize the space? Have you made any

significant modifications?

After interview, can transition to a walk around the house and talking to older adult.

**Interview Guide for older adults**

3-PART INTERVIEW

● Part 1: Receiving Care

● Part 2: Life as Older Adult

● Part 3: Context of Care

**Part 1: Receiving Care**

1. How many hours per day does your caregiver spend with you?

2. What kind of activities do you get assisted with?

3. Of these cards, which ones are you get help for? Of these, name the top 3 that are

challenging, and the 3 easiest. Explain why. [CARD SORT]

a) ADLs

i) Bathing

ii) Eating

iii) Getting dressed

iv) Toileting

v) Transferring

vi) Continence

b) iADLs

i) Medication management

ii) Pet care

iii) Managing finances

iv) Preparing meals

v) Shopping

vi) Driving or managing other forms of transportation

vii) Home maintenance

viii) Cleaning and laundry

ix) Using communication devices such as a phone

1. What aspects of your condition worries you the most?
2. What is the most meaningful part of the care that you receive?
3. In case of emergencies, what do you do?
4. Please describe the most recent encounter with a medical professional. Why? What

happened? Any recent hospitalizations?

**Part 2: Life as Older Adult**

8. Who else supports for you beyond the caregiver?

9. What gives you energy when you are feeling low?

**Part 3: Context of Care**

10. When was the last time you moved homes? If yes, how did you feel about the move?

11. Can you tell us about the space? How do you feel in the current space? What works well

and what do you think could be changed?

**APPENDIX 2: Diary study for older adults and caregivers**

**Diary study for caregiver**

**Diary study for older adults**

**APPENDIX 3: Master code list**

| **Code** | **Frequency** |
| --- | --- |
| Living situation | 83 |
| Medication Management | 76 |
| Older adult - medical care | 71 |
| Dementia/Memory | 69 |
| Mobility | 67 |
| Caregiver affect - worry | 66 |
| Caregiver - family support | 65 |
| Preparing meals | 63 |
| Caregiver Demographics | 58 |
| Caregiver employer status | 55 |
| Caregiver family | 54 |
| Paid caregivers | 51 |
| Older adult demographics | 47 |
| caregiver - education | 46 |
| Home modifications | 45 |
| Toileting | 43 |
| Care financing | 42 |
| Older adult independence | 42 |
| Care coordination | 41 |
| Falls | 40 |
| Caregiver responsibility | 38 |
| Caregiver burden | 36 |
| Hospitalization | 30 |
| Caregiver – Older adult relationship | 29 |
| Dressing | 29 |
| Older adult technology | 29 |
| Emergency | 29 |
| Proximity to family | 29 |
| Caregiver - innovation | 28 |
| Feeding | 28 |
| Showering | 28 |
| Transportation | 28 |
| Caregiver affect - fear | 26 |
| Caregiver affect - stress | 26 |
| Housekeeping | 26 |
| Older adult - dignity | 24 |
| Nursing home | 24 |
| Older adult - Food | 23 |
| Cleaning | 22 |
| Community centered | 22 |
| Routine | 22 |
| Caregiver affect - responsible | 21 |
| Older adult - agitation, panic | 20 |
| Caregiver - sleep | 19 |
| Older adult - condition | 19 |
| Older adult - religion | 19 |
| Groceries | 19 |
| Caregiver - health | 18 |
| Older adult employer status | 17 |
| Resources - Education | 17 |
| Caregiver - technology | 16 |
| Older adult - finances | 16 |
| Bathing | 15 |
| Caregiver - financial responsibility | 15 |
| Caregiver affect - frustration | 15 |
| Caregiver affect - relief | 15 |
| Cultural norms - social expectations | 15 |
| Older adult affect - worry | 15 |
| Emotional connection | 15 |
| Older adult isolation | 14 |
| Emotional care | 14 |
| Network | 14 |
| Remote care | 13 |
| Cultural norms - religion | 12 |
| Older adult affect - stubbornness | 12 |
| Caregiver - medical learning | 11 |
| Caregiver isolation | 11 |
| Older adult affect - frustration | 11 |
| Outside help | 11 |
| Quality of care | 11 |
| Caregiver affect - care | 10 |
| Caregiver friends | 10 |
| iADL - finances | 10 |
| Medical care | 10 |
| Pets | 10 |
| Caregiver affect - acceptance | 9 |
| Decline | 9 |
| Older adult - combative | 9 |
| Mental wellbeing | 9 |
| Respite care | 9 |
| Distant supportive care | 8 |
| Emotional exhaustion | 8 |
| Interdependent society | 8 |
| Caregiver affect - guilt | 7 |
| Caregiver affect - validation | 7 |
| Older adult - end of life | 7 |
| Exercise | 7 |
| Proximity to care | 7 |
| ADL - Feeding | 6 |
| Caregiver - end of life contemplation | 6 |
| Caregiver life | 6 |
| Caregiver physical impact | 6 |
| Grooming | 6 |
| ADL | 5 |
| Caregiver - Early retirement | 5 |
| Caregiver affect - calm | 5 |
| caregiver affect - helpless | 5 |
| Caregiver affect - patience | 5 |
| Caregiver affect - resignation | 5 |
| Older adult - Fear | 5 |
| Older adult - happiness | 5 |
| Older adult affect - embarrassment | 5 |
| Home care | 5 |
| iADLs | 5 |
| Caregiver affect - exhausted | 4 |
| Caregiver affect - fulfillment | 4 |
| Co-dependency | 4 |
| Cultural norms | 4 |
| Decision making | 4 |
| Older adult - pain | 4 |
| Older adult affect - loss of awareness | 4 |
| Meditation | 4 |
| Monitoring | 4 |
| Socioeconomic status | 4 |
| Temporary relievement | 4 |
| Caregiver affect - despair | 3 |
| Caregiver affect - sad | 3 |
| Diagnosis | 3 |
| Older adult affect - boredom | 3 |
| Hearing | 3 |
| iADL - Cooking | 3 |
| Physical exhaustion | 3 |
| Reducing burden | 3 |
| Caregiver affect - superior | 2 |
| Older adult affect - helplessness | 2 |
| Older adult affect - positive | 2 |
| Entertainment | 2 |
| Gender | 2 |
| Generational continuity | 2 |
| Laundry | 2 |
| Literacy | 2 |
| Medical - infection | 2 |
| Peace of mind | 2 |
| Proximity to city center | 2 |
| Severity | 2 |
| Shopping | 2 |
| Travel | 2 |
| Caregiver – Older adult - DIFF | 1 |
| Caregiver affect - "Not enough" | 1 |
| Caregiver affect - disgust | 1 |
| Caregiver affect - gratefulness | 1 |
| Caregiver affect - reliance | 1 |
| Caregiver affect - unhappy | 1 |
| Co-morbidities | 1 |
| Compulsion | 1 |
| Considerate | 1 |
| Older adult affect - nostalgia | 1 |
| Exercise | 1 |
| Health system | 1 |
| Hobby | 1 |
| Journey Map | 1 |
| Nighttime care | 1 |
| Nostalgia | 1 |
| Planning future | 1 |
| Regret | 1 |
| Second Opinion | 1 |
| Services | 1 |
| Superstition | 1 |
